# ACUTE NAD+ SUPPLEMENTATION MODULATES SYSTEMIC INFLAMMATION IN A LPS-INDUCED DELIRIUM MOUSE MODEL

**DOI:** 10.1093/geroni/igae098.3632

**Published:** 2024-12-31

**Authors:** M K Kirsten Chui, Prasanna Vadhana Ashok Kumaar, Eric Verdin, John Newman

**Affiliations:** Buck Institute for Research on Aging, Novato, California, United States; Buck Institute for Research on Aging, Novato, California, United States; Buck Institute for Research on Aging, Novato, California, United States; Buck Institute for Research on Aging, Novato, California, United States

## Abstract

Delirium is a serious neuropsychiatric condition that lacks an effective treatment intervention. A confusional state often brought on by acute illness, delirium is associated with acute inflammation and metabolic dysfunction. NAD+ is a metabolite involved in both cellular energy generation and immunomodulation, that has previously been found to promote brain health, metabolic functions, and reduce inflammation. Whether NAD+ supplementation may be beneficial for delirium has not been explored yet. In the present study, we investigate the effect of acute supplementation of NMN, a direct precursor of NAD+, in a LPS-induced acute inflammation mouse model for delirium. While NAD+ did not rescue the LPS-induced sickness behavior and metabolic dysfunction in mice, a comprehensive cytokine profile analysis did reveal rescue of plasma IFNγ levels by NMN supplementation. We also observed partial improvement on the levels of IL-12p40, LIX, and IL-17 which were sex dependent. Overall, we demonstrated that acute NAD+ boosting can lessen the LPS-induced cytokine storm in aged mice.

